# Endodontic practice amongst Nigerian dentists undergoing postgraduate training

**DOI:** 10.11604/pamj.2021.39.218.23205

**Published:** 2021-07-28

**Authors:** Shakeerah Olaide Gbadebo, Deborah Mojirade Ajayi

**Affiliations:** 1 Department of Restorative Dentistry, Faculty of Dentistry, College of Medicine, University of Ibadan, Ibadan, Nigeria,; 2 Department of Restorative Dentistry, University of Ibadan, Ibadan, Nigeria

**Keywords:** Endodontics, postgraduate training, endodontic guidelines, rubber dam, root canal treatment

## Abstract

**Introduction:**

this study aimed at finding out current practice of endodontics amongst Nigerian dentists undergoing postgraduate training (also referred to as dental resident doctors) in different institutions across the nation.

**Methods:**

a questionnaire-based, cross sectional study was conducted amongst dentists undergoing postgraduate training. Questions were asked on demographics, protocol for root canal treatment (RCT), materials employed in different stages. Opinions were also sought on satisfaction with their practice and training needs in endodontics. Data were analyzed with SPSS version 20.0 and presented as tables and charts. Significance level was set at p≤0.05.

**Results:**

ninety dentists undergoing postgraduate training (57 males and 33 females) with mean age of 34.81 ± 5.9 years participated in the study. Root canal treatment was mostly done in multiple visits in both single and multi-rooted teeth (p=0.01), only about 15% performed the procedure on multi rooted teeth. Sixty-five (72.2%) never used Rubber dam, stainless steel files were being used by 69%, step down technique of preparation by 53.9% and Sodium hypochlorite was the major irrigant (80%) used. Obturation was majorly with Cold lateral compaction technique (94%), 57.2% delayed definitive restoration for maximum of 1 week and amalgam was still the major material used for posterior teeth as reported by 62.9% of the participants. The majority (55.6%) were not satisfied with their current knowledge and practice and most were those that did not have good undergraduate training (p = 0.05).

**Conclusion:**

the practice of endodontics needs standardization across the nation as it is being advocated in other countries. There is need for hands on-training on endodontics to encourage adoption of new advances in technology, as well as improve the training of postgraduate dentists in endodontics. Also, emphasis should be placed on use of rubber dam in order to minimize the spread of infection and protect the patients from aspiration of small instruments involved in endodontic procedure.

## Introduction

Endodontics is widely practiced across the globe to alleviate pulpal pain and pathologies in order to maintain the affected tooth as a functional unit of the dental arch [1]. Many innovative concepts, techniques and instruments have been introduced in the field of endodontics for effective cleaning, shaping and obturation of infected root canals in the past few decades [2]. Root canal treatment is technically demanding and it fails when treatment falls short of acceptable standards [3]. With the development of new technologies which ranged from advances in imaging techniques for diagnosis and treatment, to instruments and materials for cleaning, shaping and obturation of root canals as well as regeneration of diseased pulpal tissue, many teeth with guarded prognosis that used to be extracted in the past can be salvaged by surgical or non-surgical endodontic treatment [4,5]. Guidelines have been formulated in the past decades reflecting an increased interest in quality assurance in endodontic procedures [5,6]. Although the viewpoints of academic teaching and endodontic societies are clear, little information is available regarding the attitude of dental practitioners towards these standards in Nigeria, and on how far the advances in endodontic technique have been incorporated into daily practice. With these guidelines, root canal treatment should be standardized across the world. This standard protocol practice is expected to be actualized in university study programs for both undergraduates and postgraduates and supervised by adequately trained specialists.

Several studies [7-11] have revealed that the majority of general dental practitioners may not be complying with the international recommended guidelines. This view, however, may be different with dentists that are undergoing or that have undergone some more special training in endodontic practice. Furthermore, it is pertinent to know what obtains among the postgraduate trainees as regards infection control during the practice of endodontics. This is important because most times aerosols are generated with use of hand-piece during drilling. Thus, adequate infection control is mandatory to prevent cross infection among patients, dentists and other paradental staff. Therefore, this study aimed at finding out current practice of endodontics amongst Nigerian dentists undergoing postgraduate training in different institutions across the nation, compared with what is practiced internationally and also to know the opinions of these postgraduate dentists in training, on their level of practice and training needs.

## Methods

This was a cross sectional study that employed self-administered questionnaire completed by resident doctors during the update course of West African College of Surgeons and residents that attended a conference of the Nigerian society for restorative dentistry (NISORD) in the same year. The update course usually draws dentists undergoing postgraduate training in institutions across the nation. To avoid duplication, the postgraduate doctors were asked to indicate if they had already completed the same questionnaire previously. The modified questionnaire [11] was distributed by the representative of the doctors. The questionnaire had 2 parts: part A asked questions on the demographic data, while part B had 36 questions that asked about protocol for endodontic treatment, materials employed in different stages of endodontics and more. Other information obtained included opinions of practitioners on their level of practice and training needs in endodontics. The conduct of the research was consistent with the declaration of Helsinki on ethical principles for research involving human subjects with ethical approval sought and obtained from UI/UCH ethical review board, as well as informed consent taken from the participants. Data were analyzed using Statistical Package for Social Sciences (SPSS) (IBM SPSS statistics for Windows, Version 23.0. Armonk, NY: IBM Corp.) Descriptive statistics were employed and results were reported as percentages/proportions and presented as tables and charts. Chi square was used to assess the relationship between undergraduate training and knowledge /practice of endodontics as postgraduate trainees. Statistical significance was set at p≤0.05.

## Results

**Figure 1:**
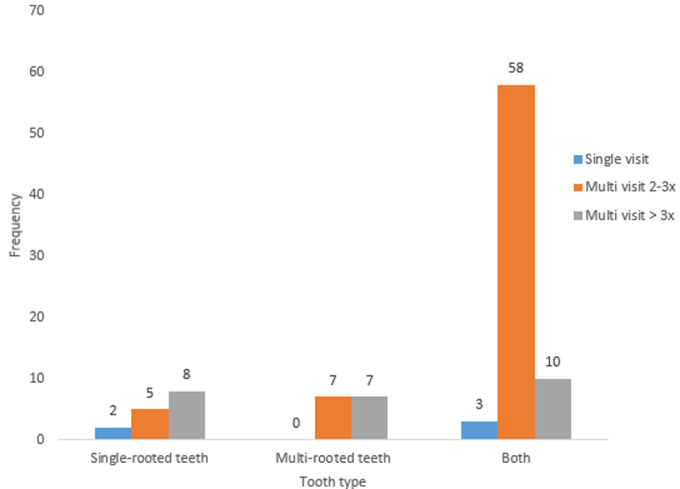
number of visits RCT was performed in various tooth types

**Figure 2:**
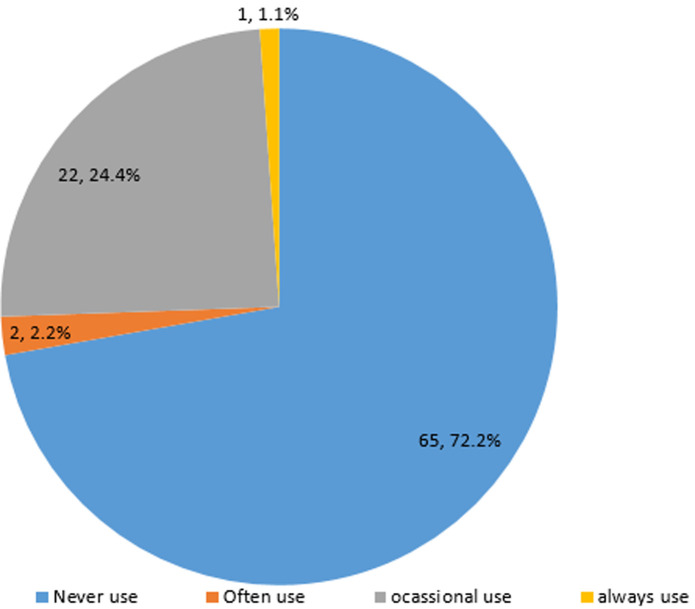
use of rubber dam during endodontics

**Figure 3:**
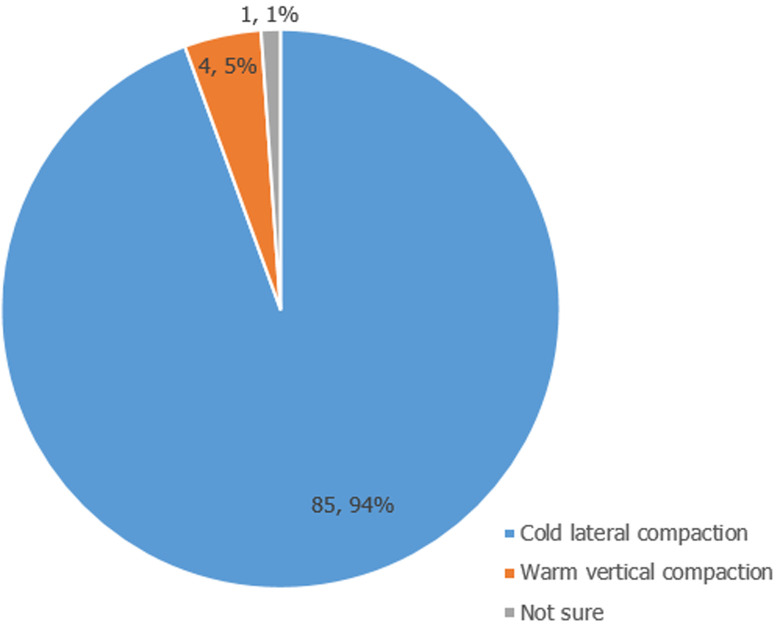
obturation techniques for endodontics employed by participants

Ninety respondents (57 males and 33 females) filled and completed the questionnaire. The mean age of participants was 34.81 ± 5.9 years. A high proportion (72; 80%) of the participants graduated between 2001 and 2010 and the majority (86.7%) work with the government (Table 1). Table 2 shows that the majority (77.8%) of participants performed an average of 5 root canal treatments (RCT) per week, and only about 15% of the participants performed the procedure on multi rooted teeth. Figure 1 shows that RCT is mostly done in multiple visits of at least 2 to 3 visits in both single and multi-rooted teeth (p=0.01). About 90% take preoperative radiograph routinely, and 92.9% still use conventional/analogue radiographs (Table 3). Rubber dam was never used, as reported by 65 (72.2%) of the participants (Figure 2). Table 3 shows that stainless steel files were being used by 69% of respondents, step down technique of biomechanical preparation was employed by a majority (53.9%), sodium hypochlorite was majorly the irrigant of choice (80%), zinc oxide eugenol-based sealer was used by a high proportion (37; 41.1%) of the participants and cold lateral compaction technique was the most commonly employed technique of obturation (94%) (Figure 3). The main temporary restorative material was zinc oxide eugenol cement and its variants as reported by 94.2% of the participants, while a high proportion (57.2%) delay the placement of the definitive restoration of access cavity for at least 24 hours to maximum of 1 week (Table 4). The majority (69.2%) nonetheless, still use amalgam for definitive restoration of access cavity in the posterior region (Table 5). The majority (55.6%) of the participants were, however, not satisfied with their current knowledge and practice of endodontics and most of these respondents were those that felt they did not have good undergraduate training on endodontics. (p= 0.05) Table 6.

**Table 1:** demographic of participants

Variable	N	%
**Gender**		
Male	57	63.3
Female	33	36.7
**Year of graduation**		
< 1990	1	1.1
1991-2000	11	12.1
2001-2010	72	80.0
>2010	6	6.7
**Place of work**		
Government hospital	78	86.7
Private hospital	10	11.1
Others	2	2.2

**Table 2:** preoperative assessment, practice and frequency of RCT

Variable	N	%
**Do patients sign consent form prior treatment**		
Yes	5	5.6
No	84	94.4
**Do you check tooth vitality prior RCT**		
Always	28	311.1
Often	20	22.2
Occasionally	37	41.1
Never	5	5.6
**Type of tooth being treated and frequency**		
Single rooted teeth	15	17.0
Multi rooted teeth	14	15.9
Retreatment	1	1.1
Both	58	65.9
**Average number of RCT per week**		
0-5	70	80.5
6-10	13	14.9
11-15	2	2.3
16-20	1	1.1
>20	1	1.1
**Do you routinely take preoperative radiograph?**		
Always	81	92.0
Often	6	6.8
Never	1	1.1
Occasionally	0	0
**Type of radiograph being used**		
Conventional/analogue	78	86.7
Digital	5	5.6
Others	1	1.1
No response	6	6.7

**Table 3:** intraoperative protocol: materials and methods in use for current endodontic practice

Variable	N	%	Variable	N	%
**Sterilization of files**			**Use of topical anaesthesia prior to injection**		
Autoclave	74	82.2	Always	Always	1.1
Cold sterilization	12	13.3	often	4	4.4
Discard after single use	1	1.1	occasionally	64	67.8
No response	3	3.3	Never	24	26.7
**Number of visits for RCT**			**Type of file being used**		
Single visit	5	5.6	Stainless steel	58	69.0
Multi-visit (2-3 visits)	60	66.7	Nickel titanium (NITI) files	17	20.2
Multi-visit (>3 visits)	25	27.8	Both	9	10.7
**Canal instrumentation**			**Use of apex locator**		
Hand instrumentation	85	95.5	Always	0	0
Rotary	3	3.4	Often	3	3.4
Both	1	1.1	Occasionally	22	25.3
			Never	62	71.3
**Extent of preparation from radiographic apex**			**Method of Biomechanical preparation**		
1-2mm	41	47.1	Step back technique	48	53.9
2-3mm	1	1.1	Crown down technique	12	13.5
0.5mm	33	37.9	Conventional	21	23.6
As far as apex	12	13.8	Hybrid	8	8.9
**Use of magnifying lens/ operating microscope**			**Type of irrigants**		
Always	1	1.1	Sodium hypochlorite chlorhexidine distilled water	64. 31	81.1 3. 3 1.2
Often	5	5.6	Hydrogen peroxide	2	2.2
Occasionally	11	12.2	Normal saline	1	1.1
Never	73	81.1	Sodium hypochlorite + normal saline	9	10.1
			No Response	10	11.1
**Sodium hypochlorite concentration**			**Number of times files are used**		
≤ 2%	19	21.1	Single use	2	2.3
5%	36	40	Multiple use	84	97.7
Others	3	3.3			
Don't know	32	35.5			
**How often is file separation encountered**			**Use of Gate Glidden burs for coronal flaring**		
Always	1	1.1	Always	2	2.3
Often	2	2.2	Often	8	9.2
Occasionally	42	47.7	Occasionally	25	28.7
Never	43	48.9	Never	52	59.8
**Intracanal medicament**			**How often master cone radiograph is taken?**		
Camphor quinine(CMCP)	10	11.6	Always	41	46.6
Formocresol	4	4.7	Often	11	12.5
Calcium Hydroxide	68	79.1	Occasionally	26	29.5
No medicament	4	4.7	Never	10	11.4
**Total no of radiograph taken for RCT**			**Type of sealer being used regularly**		
2	2	2	Calcium hydroxide based	16	19.0
3	55	64.0	Zinc oxide Based	37	44.0
4	2	33.7	Resin based	29	34.5
			All of the above	2	2.4
**Prescription of systemic antibiotics during RCT**					
Calcium hydroxide based	9	10			
Often	16.7	16.7			
Occasionally	55	61.1			
Never	9	10			
No response	5	5.6			

**Table 4:** coronal restoration on completion of RCT

Variables	N	%
**Type of temporary material**		
Conventional zinc oxide eugenol	69	80.2
Cavit	11	12.8
Intermediate Restorative material	1	1.2
Glass ionomer cement	2	2.3
Others	3	3.5
**Time of placement of definitive coronal restoration**		
Immediately post obturation	33	39.3
24 hours post obturation	4	4.8
1week post obturation	44	52.4
Not Specific	3	3.6
**Time of placement of protective crown in posterior teeth post RCT**		
1 week	6	6.7
2 weeks	21	23.3
3 weeks	45	50.0
No consideration for crown	18	20.0

**Table 5:** type of material for definitive coronal restoration

Type of definitive coronal restoration	Anterior		Posterior	
	N	%	N	%
Amalgam	1	1.1	54	69.2
Composite	76	84.1	16	20.5
GlassiIonomer cement(GIC)	2	2.2	2	2.6
Composite + GIC	2	2.2	0	0
Amalgam + composite	0	0	6	7.5
Not specific	9	10	0	0

**Table 6:** resident dentists satisfaction with current endodontic practice

Variable	Was your undergraduate endodontic training adequate			X2	P Value
	Yes		No		
Are you satisfied with your current endodontic practice	Yes	21	19	3.86	0.05
	No	16	34		

## Discussion

The participants in this study were postgraduate resident doctors undergoing training in different institutions across six geopolitical zones of Nigeria, unlike the study by Udoye *et al*. [7] on endodontic practice in Nigeria, which reported on dentists practicing in the Southwest of Nigeria only. This therefore, makes our study participants more representative of the country to give valid and valuable information on the current status of endodontic practice in a sub population of Nigerian dentists and may also give a better view of the practice of non-surgical endodontics i.e. root canal treatment (RCT), across the country. The majority of the participants (being postgraduate doctors undergoing training) work with the government unlike the participants involved in a Turkish survey [1], where most respondents (73.5%) were randomly selected dental practitioners practicing at private dental clinics. Also, a high proportion (76.7%) of the participants graduated between 2001 and 2010 with an average of 6-10 years of experience; similar to studies in Jordan [8] and Iran [9] where 53.4% and 82.6% of the participants respectively had an average of 0 to 10 years of experience. This is, however, contrary to a Turkish study [1] where the majority (71.5%) of the participants graduated between 1981-2000 with more than 10years experience and Gupta *et al*. [3] where only 29% had 6-10 years of experience. The majority (77.8%) of the participants in this study performed RCT on an average of 5 teeth per week. Also, only about 15% of the participants performed the procedure on multi-rooted teeth unlike in Kathmandu (India) [12] report in which 97% of the respondents worked on multi rooted teeth. The lower percentage of participants working on the posterior teeth may be due to poor attitude of many Nigerian patients towards conservative treatment. Also, many patients would rather opt for extraction due to their unaffordability of cost of RCT. According to European Society of Endodontology (ESE) guidelines, [5,6] written information and informed consent should be obtained from patient prior to root canal treatment. This study, however, showed that the majority (93.3%) of the participants treat patients based only on verbal consent which is usually a practice in this environment for supposedly a less /non-invasive procedure. It is better, however, to have a written document in order to avoid litigation.

Autoclaving as claimed by 82.2% is the major means of sterilizing instruments and files. Though the use is higher in this study, it is also a major mode of sterilization in a study by Gupta *et al*. [3] where 48% claimed the use of autoclave. By standard of sterilization, autoclaving is the most rapid and effective method for sterilizing. It is dependable, economical and the sterilization outcome is verifiable [13]. This study showed that a high percentage (90%) of the participants take pre-operative radiographs according to recommendations [14]. This finding is higher than what was reported by Gupta and Rai [3], Mehta *et al*. [15] and Küçükkaya *et al*. [1], that recorded 87%, 81% and 60.3% respectively. However, conventional radiography is what the majority make use of in this study though advanced imaging techniques ranging from digital radiography to computer-based tomography [2] are now available for better diagnosis and treatment planning. The use of conventional radiograph is possibly due to the poor health financing in the country which has led to unavailability of technologically advanced facilities. It is not surprising that the majority of the participants (72.2%) in this study claimed not to use rubber dam routinely, as previously documented by other studies [1,9-16]. While only 1.2% and 9% claimed they use in study by Udoye *et al*. [7] and Iqbal *et al*. [17] respectively, a United Kingdom study [18] reported that about a quarter of respondents routinely used rubber dam during root canal therapy. Nevertheless, in America [19] and Flemish population [20], the percentages of dentists that claimed routine use of Rubber Dam were 59% and 56% respectively.

Though new modifications are available to overcome the possible disadvantage (in application) of the old and conventional rubber dam kit, yet majority still do not use it. Reason for no use may include extra cost of rubber dam, lack of training, unavailability and unacceptability as observed by Gupta and Rai, [3] and Udoye *et al*. [7]. The major substitute for this restorative dentistry (RD) is cotton wool roll and gauze with regular suctioning using high volume suction [21]. However, the use of RD will go a long way in preventing transfer of infection between patients and doctors. The hallmark of RCT is the cleaning, shaping and obturation of the canal with resultant fluid tight seal [22]. This study showed that a great majority (94.4%) use hand instrumentation, a high number (69%) makes use of stainless steel (SS) files in shaping the canal and about half of the participants (53.3%) employ stepback technique of root canal preparation. This confirms that the use of hand instruments is still popular amongst resident dentists as observed in other studies such as Shresta *et al*. [12] who observed that 88% of respondents use SS K files while only 12% use rotary instruments. Also, some researchers [9,17] have reported higher use of SS K files with hand instrumentation as common practice amongst dentists in their countries. On the contrary, Mozayeni *et al*. [23] in their study found that the majority (98.4%) of endodontic specialists use rotary instruments while only half (50.6%) of the general dentists make use of it. This further confirmed the higher use of rotary instruments among endodontists when compared with general practitioners. Advantages of rotary instruments which are based on nickel titanium (NiTi) include their super elasticity with their advanced design for effective and safe instrumentation of narrow and curved root canals using low torque hand pieces. However, the non-popular use of these NiTi rotary instruments for canal shaping has been reported to be due to lack of adequate education and hands-on training, no perceived advantage of NiTi, inexperience, unavailability of NiTi instruments and easy availability of hand instruments [7,23]. The extra cost required to purchase the rotary instruments and the corresponding gutta percha points to be used for obturation of the mechanically prepared canals may also be contributory.

Also, with excessive use of hand files, separation of these instruments within the canal is experienced often. As observed in this study, over 40% of the participants experienced instrument separation and this could be due to over-use. The technique of preparation of canal that involves the early coronal flaring, for example, crown-down technique has been found to produce a better shape, prevention of transportation of debris and micro-organisms to the apex, and enhanced penetration of irrigating solution. However, this study like many others [7-9], observed that the majority of Nigerian dentists still make use of stepback technique despite the several advantages of crown-down technique. Furthermore, some of the respondents (23.3%) in this survey still use standardized technique as also shown by another study [17] where 47.5% use this same technique. A major disadvantage of this technique is over preparation resulting in elliptically shaped defect at the end-point of preparation [17]. The procedures involved in RCT can be completed in either single or multi-visits. Despite the advantages attributed to single-visit, which include eliminating inter-appointment microbial contamination and flare-ups caused by coronal leakage or loss of the temporary seal, reduction of fear and anxiety in the apprehensive patients, only 5.6% claimed they do single visit RCT and mostly on single rooted teeth. However, an evidenced based review study [24] has shown that there is no substantial difference in the success rate of single and multiple appointments´ cases. Though encouraged based on advantages, case selection for single-visit must be well done to avoid complications and unnecessary retreatment. Working length determination is an important step in the endodontic treatment and use of apex locator has been found to improve and give accuracy in locating the apical foramen [25]. In this study, the majority (68.9%) of the participants claimed they had never used apex locator. This result is in accordance with what was reported in other studies [17,26]. Only about 24% claimed they use it occasionally unlike in the report by Udoye *et al*. [7] which had no record of use at all and higher than 7% use reported by Iqbal *et al*. [17]. Possibility of unawareness of its importance and unavailability in some hospitals may be the reason for this result. It is, however, logical to combine the use of electronic apex locator and radiographs for effective working length determination.

The irrigant of choice in this study was majorly sodium hypochlorite which is similar to what has been reported by several studies [1,3,12-17]. Sodium hypochlorite acts as a lubricant during instrumentation, possesses a broad-spectrum antimicrobial activity against endodontic microorganisms and has ability to dissolve organic material. It has been documented to be very effective as bactericidal but has low organic dissolving ability which can be overcome by combining it with other chelating agents [27]. It is also cheap and readily available. Though in this study, some reported not knowing the percentage concentration they use, the majority (61.1%) use less than 5% concentration. This may be due to the non-use of rubber dam (which is a documented reason for use of low concentration of sodium hypochlorite) in order to prevent the possible caustic effect it may have on soft tissues [27]. This study shows over-use/inappropriate use of antibiotics with majority prescribing antibiotics for all cases of endodontics which is in agreement with other researchers [12]. The use of antibiotics in endodontics should be limited to when there is associated abscess and systemic symptoms [28]. Additionally, in order to prevent potential risk of adverse effects following systemic application, and the ineffectiveness of systemic antibiotics in necrotic teeth and the peri-radicular tissues, the local application of antibiotics as irrigant, has been suggested to be a more effective mode of delivery in endodontics [28]. The appropriate use will also reduce risk of drug resistance. Intra canal medicament is importantly used in multi-visit RCT to further reduce micro-organisms within the canal. The majority of the participants in this study use calcium hydroxide as intracanal medicament in accordance to what has been reported by other researchers [9,26]. Calcium hydroxide is said to be the gold standard for intracanal medicament especially in infected canals [29]. However, residual calcium hydroxide can shorten the setting time of zinc oxide eugenol-based endodontic sealers [29]. It may also interfere with the seal of the root filling and compromise the quality of treatment. In contrary to this study´s result, Iqbal *et al*. [17] reported the use of formocresol as the major intra canal medicament. Also, Udoye *et al*. [7] reported the use of eugenol as the preferred medicament. This difference may be due to study area and study participants being from just a part of the country. Formocresol has mutagenic effect [30] and its use should be discouraged. Eugenol on the other hand has anodyne effect but it is allergenic [31].

Cold lateral compaction technique has been reported as the most frequently employed technique of obturation by several studies [3,7,9,17-20] including this present one. It is a technique that is acknowledged universally and has been found to be simple and easy to perform. However, warm lateral compaction has the advantage of sealing lateral and accessory canals.Zinc oxide eugenol-based sealer is employed by a high proportion of the participants, followed by resin-based sealer e.g. AH26. This result is in accordance with other researchers [3,12,18], and contrary to a study [20] where resin-based sealer was majorly used, but only by about 30% of the participants. Zinc oxide based sealer has antimicrobial activity and is slow setting, giving an ample working time which is an advantage [32]. However, it shrinks on setting, is soluble in tissue fluids, and can stain tooth structure [32]. Due to allergy to eugenol by some people, non-eugenol- based sealers and other sealers with better sealing ability were introduced. These include: resin- based sealer; AH 26, AH plus; bioceramic; and epiphany which produces a new bond (monoblock) between a new core obturating material Resilon and the canal walls [32]. During multi- visit RCT, the material of choice for temporary restoration of access cavity must provide a high quality seal of access cavity to prevent microbial contamination of the root canal. The majority (69%) of the participants use the conventional zinc oxide (ZnOE) material while only 11% make use of cavit. Cavit is a zinc oxide-based material which has been found to be more resistant to leakage when compared with other materials [33]. In the same vein, the final access cavity restoration should provide adequate coronal seal, with no marginal microleakage and provide adequate and acceptable masticatory and aesthetics functions. Most participants in this study use composite resin material for anterior teeth restoration (Table 5) as also observed by Guptal *et al*. [3] this may be due to its excellent aesthetic quality and good strength. Amalgam was the major material for posterior teeth restoration. However, due to the improvement in material science and adhesive dentistry, composite resin too can be used in posterior teeth with good result.

Almost half (48.9%) of the participants in this study restore the access cavity a week after obturation. It is recommended by the American Association of Endodontists (AAE) [34] that restoration should be placed as soon as possible after RCT and even in teeth for extra coronal restoration, whenever possible, bonded core buildup should be placed at the obturation appointment. The cusps of root filled posterior teeth are to be protected against fracture after RCT by placement of at least the mesial-occlusal-distal (MOD) onlay, endocrown and full crown [35]. However, use of crown to protect these types of teeth is common as observed by Gupta *et al*. [3]. This is also the practice by Nigerian dentists but majority (50%) place the crowns 3 weeks post obturation. This study like some others [1,3,12] also established the fact that undergraduate training in endodontics may be deficient as shown by more than 60% of the participants who claimed they were not satisfied with their undergraduate training and the majority of these people are presently still not satisfied with their current practice (p=0.05) and would appreciate more training especially hands-on in the specialty of endodontics. The study also showed more improvement in endodontic practice in the country in contrary to what has been earlier documented [10]. Relatively more awareness about dental treatment, need for conservative management, increase in number of dental schools in the country, and possibly mandatory endodontic training at junior level for all postgraduate dentists in training may be responsible.

## Conclusion

This study showed that some practices are in line with the recommendations for endodontic practice. However, the practice of endodontics in the country needs to be standardized across the nation as it is being advocated in some other countries, for effective management of the patients. There is also a need for continuing medical education (CME) and hands on-training on endodontics for dentists, to encourage adoption of new advances in technology, as well as improve postgraduatetraining in endodontics. Also emphasis should be placed on use of rubber dam in order to minimize the spread of infection and protect the patients from aspiration of small instruments involved in endodontic procedure.

### What is known about this topic

- Studies have reported that most general dental practitioners have low adherence to use of international guidelines for endodontics;
- Though better amongst studied group compared to what is known with general dental practitioners, use of latest techniques and materials in endodontics is poor;
- Use of rubber dam is not commonly practiced both in the studied group and internationally.

### What this study adds

- This study has shown that undergraduate training in endodontics may be insufficient as many of the respondents were not satisfied with it. Thus, need to intensify endodontic training at undergraduate level;
- Study observed the non-use of rubber dam for endodontics among the studied group. Therefore, there is need to lay emphasis on use of rubber dam as a standard practice of endodontic treatment;
- Also, the study showed the need to optimize endodontic training at postgraduate level with hands-on for better knowledge in endodontics.
